# Commonly Used Analytical Tools and Methods for the Discrimination of Honey Types Based on Volatile Organic Compound Profiles

**DOI:** 10.3390/molecules31040638

**Published:** 2026-02-12

**Authors:** Gulzhan Khamitova, Simone Angeloni, Lazzat Karasholakova, Giovanni Caprioli

**Affiliations:** 1 Research Institute of Biotechnology and Ecology, Zhetysu University Named After I. Zhansugurov, Zhansugurov Street, 187a, Taldykorgan 040009, Kazakhstan; gulzhan.khamitova@unicam.it (G.K.); lyazzat.karasholakova@gmail.com (L.K.); 2 Chemistry Interdisciplinary Project (ChIP), School of Pharmacy, University of Camerino, Via Madonna delle Carceri, 62032 Camerino, Italy; simone.angeloni@unicam.it

**Keywords:** honey, aroma, volatile organic compounds, VOCs, analytical instruments, GC-MS

## Abstract

Honey is a complex natural product with nutritional and therapeutic properties that depend on the diversity of its chemical composition, which includes volatile organic compounds (VOCs). VOCs in honey are key indicators of its botanical and geographical origin, as well as its quality and authenticity. This review provides a comprehensive overview of the analytical instruments and methods used for the identification and quantification of VOCs in different types of honey. Techniques such as headspace solid-phase microextraction (HS-SPME) are used for VOC extraction, and gas chromatography coupled with mass spectrometry (GC-MS) and electronic nose (e-nose) systems for honey analyses, as well as their advantages, limitations, and applications and challenges related to VOC analysis, such as for different types of honeys, their aroma profile, compound variability, and data interpretation, are also discussed. By summarizing recent advancements in analytical methodologies, this review provides an overview of the analysis of VOCs for authentication and research purposes in honey production and processing.

## 1. Introduction

Honey is a natural sweetener widely appreciated for its diverse aroma and flavor profiles, which are primarily influenced by its botanical and geographical origins [1,2,3,4]. The volatile organic compounds (VOCs) present in honey contribute significantly to its characteristic scents, ranging from floral and fruity to herbal and woody notes [5,6,7,8,9,10,11,12,13,14,15]. Honey can be classified based on its entomological origins, for example, *apis* and *stingless bees*, and can be classified based on its nectar source into two primary categories: monofloral and multifloral. Monofloral honey originates predominantly from a single plant species, resulting in a distinct and consistent volatile organic compound (VOC) profile that reflects its botanical origin. In contrast, multifloral honey is derived from mixed floral sources, leading to a more complex and variable VOC composition. Stingless bee honey, produced by stingless bees (*Meliponini* species), often exhibits unique VOC signatures due to differences in bee physiology and foraging behavior. Each honey type possesses characteristic aroma profiles influenced by its specific VOC composition, which in turn affects its sensory and potential bioactive properties [7,16,17,18].

### Aroma Profile of Honey

The aroma profile of honey is consistently highlighted as a key attribute influencing product quality and attractiveness to consumers. Variations in honey aroma volatiles are largely shaped by their geographical origin and the floral sources contributing to nectar production, as well as the harvesting and processing techniques used. Volatile organic compounds are generally responsible for the unique olfactory characteristics of honey. They include aldehydes, ketones, esters, terpenes, furans, and alcohols. These compounds not only define the sensory properties of honey but also serve as markers for botanical and geographical authenticity. The variation in VOC composition is largely determined by nectar sources, processing conditions, and environmental factors [19,20,21,22,23].

The VOCs present in honey exhibit significant diversity, influenced by factors such as floral origin, climatic conditions, and geographical location [24]. Common VOCs identified in honey samples include terpenes (e.g., limonene, pinene, and cineole), which give citrus, herbal, and resinous notes, as well as aldehydes (e.g., benzaldehyde, hexanal, and lilac aldehydes) that contribute with floral, almond, or nutty aromas [5,7,25,26]. Additionally, esters such as ethyl octanoate and ethyl acetate enhance fruity and sweet characteristics, while furans and phenolic compounds (e.g., furfural and phenylethyl alcohol) introduce caramel, woody, and floral tones. Furthermore, sulfur-containing compounds like dimethyl trisulfide and dipropyl disulfide are associated with medicinal or slightly pungent odors [27,28,29]. This complex VOC profile underscores the intricate relationship between honey’s chemical composition and its sensory properties, which vary widely across different honey types [30,31,32,33,34,35,36,37,38].

Among the most abundant chemical classes of volatile compounds in honey are aldehydes, alcohols, phenolic volatiles, terpenoids, hydrocarbons, and esters [19,20,21,22,23], though the exact composition varies depending on the botanical origin. Previous studies have shown that volatile compounds are associated with specific honey types. For instance, Italian and Greek chestnut honeys have been shown to contain nonanal and cis-linalool oxide [2-[(2S,5R)-5-ethenyl-5-methyloxolan-2-yl] propan-2-ol], along with various benzene derivatives and phenolic compounds [39]. In Spanish citrus and honeydew honeys, benzaldehyde, benzeneacetaldehyde, and phenylethyl alcohol are among the distinguishing volatiles reported. Furthermore, key markers identified in pine, fir, citrus, thyme, honeydew, and multifloral honeys from Mediterranean zones and Brazil include benzaldehyde, benzeneacetaldehyde, octanal, nonanal, decanal, and several lilac aldehyde isomers [8,9]. Additionally, α-pinene, terpinolene, 2-phenylacetate, and other volatiles have been proposed as provenance markers for Argentinean honeys [10,11,12,13].

VOCs in honey samples have been widely researched with cutting-edge analytical instruments. This review provides a common applied analysis of honey VOCs from various global sources, their corresponding aroma profiles, and the floral origins influencing their compositions, and discusses more detailed analytical techniques for studying VOCs in honey types.

## 2. Volatile Organic Compounds (VOCs) Analysis in Honey

The choice of the most appropriate analytical technique is a very important phase during food analysis, including volatile compound analysis. Generally, the choice of sample preparation, instrument selection, and methodology to follow is driven by several parameters such as the experiment objective (targeted, untargeted, etc.) and the analytes’ physical–chemical characteristics, their contents, and sample type. Therefore, the current subsection provides a detailed description of the main analytical procedures adopted for VOC analysis in honey and honeydew honey. Google Scholar was used for article screening (of articles published between January 2015 and January 2025) by using the following terms: “honey volatile compounds”, “honey VOC”, “honey aroma compounds”, “honey GC-MS”, “types of honey” and “chemical composition of volatile organic compounds in honey”. Articles reporting any type of volatile compound analysis (qualitative, semi-quantitative and quantitative) in floral and honeydew honey were selected. On the other hand, duplicate records, review articles, and articles not with full access were excluded. In the end, 120 articles were included, with 46 scrutinized in full detail, as summarized in Table 1, which reports the main analytical strategies adopted in relation to the type of sample, the extraction method, and the used instrument. Generally, the analysis of VOCs in honey is performed for their essential role in honey flavor and their potential applications in botanical and geographical authentication [27,39,40,41,42,43,44]. In fact, rapidly skimming the “type of sample” column in Table 1, it is noticeable that several different botanical and geographical honey sources have been studied. Volatile compounds have also been investigated as markers of shelf-life and adulteration [16,45,46,47,48,49,50,51]. The following subsections are focused on different extraction methods and instruments used (Table 1).

### 2.1. Extraction Methods

The most adopted extraction procedure for volatile compounds in honey is headspace solid-phase microextraction (HS-SPME), as shown in Table 1. This technique, introduced for the first time by Pawliszyn [47], is characterized by the diffusion of volatiles in the headspace of a closer vial, in which the sample is located. Then, an absorbing fiber is introduced in the headspace and volatiles are absorbed onto an SPME fiber coating [52,53]. This technique does not require the use of solvents or purification steps and thus, it allows you to save time. Three types of coating fiber, i.e., divinylbenzene/carboxen/polydimethylsiloxane (DVB/CAR/PDMS), were the most used for VOC analysis in honey (see the “additional information” column of Table 1), likely due to their wide range of volatile extraction capacity. Just few papers have reported the use of PDMS/DVB and polyacrylate coatings [42,54,55,56,57,58]. Another common method for the extraction of volatile compounds is liquid–liquid extraction assisted or not with ultrasound (Table 1). Among the various solvents, dichloromethane and a mixture of pentane with diethyl ether (1:2, *v*/*v*) is generally employed; successively, the sample is concentrated by distillation and analyzed [59,60,61,62,63,64]. Some researchers have proposed alternative extraction methods for VOCs in honey such as dynamic headspace analysis (DHS) with the in-tube extraction dynamic headspace Tool (ITEX-DHS) [65], HS-mini-SPME [66], stir bar sorptive extraction (SBSE) [67], or more traditional approaches, e.g., hydrodistillation [68,69]; these are niche approaches that are not very widespread. Once extracted and purified, samples composed of mixtures of volatile compounds are generally separated and detected by gas chromatographic instruments.

### 2.2. Methods and Instruments

The GC-MS system is the most-used analytical technique for volatile compound analysis in honey both for analyte screening and quantification. After volatile extraction by the SPME approach, capillary columns with a stationary phase at low polarity or that are nonpolar, such as HP-5MS, DB-5MS or Rtx-5MS, are commonly installed on the gas chromatographer (“additional information” column of Table 1) for compound separation. The mass spectrometer detector hyphenated to GC is a low-resolution system, such as a single quadrupole, used in full SCAN acquisition modality for screening honey volatile profiles. On the other hand, SIM (Selected Ion Monitoring) acquisition is employed for quantitative studies in targeted analysis. Other analytical instruments such as GC-MS/MS triple quadrupole [69], GC-QTOF [70,71], GC-O [72,73], GC-IMS [74,75], GC-FID [59,60,61,63,64], and GCxGC-MS [76,77,78,79] are selected for specific purposes, for instance, quantitative studies, enantiomeric distribution, odor-active compound analysis, and honey discrimination. For the analysis of volatile compounds released by stingless bee honey, Wei et al., 2016 [70] and Hassan et al., 2020 [71] proposed using long-optical-path Fourier Transform Infrared Spectroscopy (FTIR). They found ethanol, methanol, methane, ethyl acetate, water vapor, and carbon dioxide in the headspace of the sample container; however, this is a particular approach for VOC analysis. Panseri et al., 2023 [72] studied the VOC and aroma profile of *Robinia pseudo-acacia* L. honey at different storage temperatures during its shelf life using GC-MS and electronic-nose (e-nose). E-nose is a powerful tool for non-destructive quality monitoring of agricultural and food products, and this device is compatible with traditional analytical methods and the human olfactory system [74].

In summary, the profile of volatile compounds in honey is generally evaluated using gas chromatography instruments coupled with mass spectrometers. HS-SPME with DVB/CAR/PDMS fiber is the primary extraction technique, while analyte separation is usually performed on a non-polar column. Finally, identification is achieved by acquiring spectra in SCAN mode and comparing them with libraries. Semiquantitative analysis is preferred for assessing analyte abundance. Modern analytical techniques, such as two-dimensional gas chromatography (GCxGC), high-resolution mass spectrometry, and ion mobility spectrometry (IMS), are increasingly adopted for their high capabilities in separating very similar analytes in complex matrices and in thoroughly characterizing the volatile profile.

### 2.3. Comparison of the Most Used Analytical Technologies

The HS-SPME-GC-MS system is the most-used technology for volatile and semivolatile organic compound analysis in complex matrices [61]. In foods analysis, this technique is generally applied for studying aroma, off flavor and volatile toxic compounds. While HS-SPME-GC-MS is the most used methodology for volatile screening in foods and in honey (qualitative analysis), the use of this technique for quantitative study is still not as widespread as it should be but will find wider application for its sensitivity, speediness, and simplicity of use. The fiber (SPME) is the most commonly used microextraction technology followed by SBSE, which is preferred just for semivolatile compounds [62]. The GC-MS/MS triple quadrupole was used for accurately quantifying some characteristic volatile compounds and could be used for distinguishing specific monofloral honey [68] or for detecting toxic volatile compounds [76]. On the other hand, the high-resolution analyzer (GC-QTOF) is preferred for its high capacity to characterize the volatile profile of honey for authenticity, traceability, quality control, storage, and processing studies [65,66]. An innovative analytical approach is the hyphenation of gas chromatography with ion mobility spectrometry (GC-IMS), which works at atmospheric pressure and does not require sample pretreatment. This technique combines the separation capacity of gas chromatography and ion mobility, which separates ions according to their drift behavior in a buffer gas under the influence of an electrical field. Therefore, this analytical system is a 2D approach that combines retention time and drift time, generating a true orthogonal separation with high capacity for volatile fingerprinting [79,80]. Moreover, this technique has the advantages of high resolution, easy operation, and analysis speed (a few minutes) but, until now, no comprehensive database for volatile identification has been developed [74]. Nowadays, HS-GC-IMS is used for the classification and adulteration of different honeys, classification between organic and traditional honey, and distinguishing honey originating from different flowers [81]. Another modern system is two-dimensional gas chromatography–mass spectrometry (GCxGC-MS), which has been applied in numerous fields of food sciences such as authenticity, contaminants, sensory science, food composition, and food-omics. GCxGC-MS systems have the capability to clearly visualize the total sample composition and all the separated compounds and their different classes, making this technique suitable for profiling/untargeted analysis [69,77]. All of the above-mentioned analytical techniques give qualitative and/or quantitative data on volatiles, but for studying odor-active compounds (aroma-active compounds) in foods, a different tool is required, such as GC-O. GC-O is a type of general GC in which a split port is installed at the end of the capillary column and some of the flow enters the detector (FID or MS) and the rest enters the sniffing port, also called the olfactometry port. This instrument permits the study of volatiles mainly responsible for food aroma, especially when a mass spectrometer is hyphenated, but it requires trained and experienced sniffers and multiple analyses for obtaining reliable data [82]. On the other hand, the e-nose is a powerful tool used in industry and academic sectors for process inspection, freshness inspection, and production analysis, and it does not require sample pretreatment or food destruction. It mimics the human olfactory system, and it can be used for evaluating the entire flavor profile of the food. On the contrary, the GC-MS system requires sample pretreatments, but it has a greater ability for volatile qualification and quantification, especially for compounds at low concentrations [82].

**Table 1:** Methods and instruments used in honey analyses.

No	Title	Type of Sample	Extraction Method	Instrument	Additional Information	Year Ref.
1	Volatile and non-volatile/semi-volatile compounds and in vitro bioactive properties of Chilean Ulmo (*Eucryphia cordifolia* Cav.) honey	Ulmo honey from Chile	HS-SPME	GC-MS	Fiber: DVB/CAR/PDMS; column: Rtx^®^-5MS (5% diphenyl/95% dimethyl polysiloxane, 30 m × 0.25 mm with a 0.25 μm film thickness); acquisition SCAN mode, *m*/*z* 20–550.	2017 [83]
2	A decisive strategy for monofloral honey authentication using analysis of volatile compounds and pattern recognition techniques	Different types of honey (number of samples: 89)	HS-SPME	GC-MS	Fiber: DVB/CAR/PDMS; column: DB-5MS (cross-linked 5% PHME PH siloxane) (60 m × 320 μm i.d., ×1 μm film thickness); acquisition SCAN mode, *m*/*z* 50–550.	2020 [81]
3	Stability of volatile compounds of honey during prolonged storage	Honey samples from Brazil	HS-SPME	GC-MS	Fiber: DVB/CAR/PDMS; column: HP-5MS (30 m × 0.25 mm × 0.25 μm).	2020 [42]
4	Volatile compounds of five types of unifloral honey in Northwest China: Correlation with aroma and floral origin based on HS-SPME/GC–MS combined with chemometrics	Fifty-six samples of honey from China	HS-SPME	GC-MS	Fiber: DVB/CAR/PDMS; column: HP-5MS (60 m × 0.25 mm × 0.25 μm); acquisition SCAN mode, *m*/*z* 50–350.	2022 [44]
5	A targeted chemometric evaluation of the volatile compounds of *Quercus ilex* honey in relation to its provenance	Thirty-four *Quercus ilex* honey samples from Greece	HS-SPME	GC-MS	Fiber: DVB/CAR/PDMS; DB-5MS (cross-linked 5% PH ME siloxane) capillary column (60 m × 320 μm i.d., × 1 μm film thickness); acquisition SCAN mode, *m*/*z* 50–550.	2022 [82]
6	Study on honey quality evaluation and detection of adulteration by analysis of volatile compounds	Honey samples (*n* = 76) were analyzed	HS-SPME	GC-MS	Fiber DVB/CAR/PDMS; HP-5MS column (30 m × 250 μm × 0.25 μm).	2017 [16]
7	Comprehensive study of volatile compounds of rare *Leucosceptrum canum* Smith honey: Aroma profiling and characteristic compound screening via GC–MS and GC–MS/MS	Nine types of monofloral honeys (China)	HS-SPME and liquid extraction	GC-MS and GC-MS/MS	Fiber: DVB/CAR/PDMS; HP-5 capillary column (30 m × 0.25 mm × 0.25 μm); acquisition SCAN mode, *m*/*z* 29–450 for GC-MS. Injection volume: 2 µL; HP-5 capillary column (30 m × 0.25 mm × 0.25 μm); acquisition MRM mode for GC-MS/MS.	2023 [69]
8	Aromatic profiles and enantiomeric distributions of volatile compounds during the ripening of Dendropanax dentiger honey	Dendropanax dentiger honey (DDH) samples from China	HS-SPME	GC-QTOF and GC-TOF	Fiber: DVB/Carbon WR/PDMS; capillary column: HP-5MS UI (30 m × 0.25 mm × 0.25 μm); acquisition mass scan range of 45–550 *m*/*z* for volatile compounds. Fiber: DVB/CAR/PDMS; CycloSil-B column (30 m × 0.25 mm × 0.25 μm); acquisition *m*/*z* scan range of 33–600 for enantiomeric volatile compounds.	2024 [70]
9	Characterization of Evodia rutaecarpa (Juss) Benth honey: volatile profile, odor-active compounds and odor properties	Eight Evodia rutaecarpa (Juss) Benth honey samples from China	HS-SPME	GC-QTOF	Fiber: SPME arrow divinylbenzene/carbon wide-range/polydimethylsiloxane; capillary column: HP-5MS UI (30 m × 0.25 mm × 0.25 μm); full scan mode, acquisition mass range *m*/*z* 45–550.	2023 [71]
10	Response surface methodology to optimize the isolation of dominant volatile compounds from Monofloral Greek Thyme honey using SPME-GC-MS	Eighty thyme honey samples	HS-SPME	GC-MS	Fiber: divinylbenzene/carboxen/polydimethylsiloxane (DVB/CAR/PDMS); capillary column: Restek Rtx-5MS (30 m × 0.25 mm i.d., 0.25 µm film thickness); acquisition scan at 35–650 *m*/*z* mass range.	2021 [84]
11	Headspace volatile compounds fluctuations in honeydew honey during storage at in-house conditions	Honeydew honey from Greece	HS-SPME	GC-MS	Fiber: DVB/CAR/PDMS; capillary column: DB-5MS (cross-linked 5% PH ME siloxane, 60 m × 320 μm i.d., ×1 μm film thickness).	2022 [85]
12	Volatile organic compounds of Thai honeys produced from several floral sources by different honey bee species	Thai honeys produced from five different floral sources by three species of honey bees	HS-SPME	GC-MS	Fiber: SPME divinylbenzene/carboxen/polymethylsiloxane; capillary column: HP 5MS (30 m × 0.25 mm × 0.25 μm); acquisition SCAN mode, *m*/*z* 24–360.	2017 [86]
13	Honey: Determination of volatile compounds, antioxidant and antibacterial activities	Honey samples were obtained directly from beekeepers in Malatya (Turkey)	HS, not clear	GC-MS	DB-5MS column (30 m × 25 mm and 0.25 μm film thickness).	2021 [87]
14	Characterization of Botanical Origin of Italian Honey by Carbohydrate Composition and Volatile Organic Compounds (VOCs)	Forty-eight samples of Apis mellifera honeys from northeastern Italy	HS-SPME	GC-MS	Fiber: divinylbenzene/carboxen/polydimethylsiloxane; VF-WAXms column (30.0 m × 0.25 mm I.D. × 0.25 µm film thickness).	2022 [88]
15	Differentiation of Monofloral Honey Using Volatile Organic Compounds by HS-GCxIMS	Fifty-eight honey samples with six different botanical origins	HS	GCxIMS and GC-MS (identification)	Column for GCxIMS: 15 m × 0.53 mm × 1 µm MXT-5; detection with a drift tube of 15.2 mm (diameter) and 98 mm (length); ionization by 3H-source at 45 °C; nitrogen drift gas flow rate of 150 mL/min; and field strength of 500 V/cm. Capillary column for GC-MS: HP-5MS UI (30 m × 0.25 mm × 0.5 µm); acquisition scan range of 32–300 *m*/*z*.	2022 [74]
16	Monitoring Volatile Organic Compounds and Aroma Profile of Robinia pseudoacacia L. Honey at Different Storage Temperatures during Shelf Life	Robinia pseudoacacia honey from Italy	HS-SPME for GC-MS	GC-MS and e-nose (electronic nose)	Fiber: Divinylbenzene/Carboxen/polydimethylsiloxane (CAR/PDMS/DVB); column: Rtx-Wax (30 m × 0.25 mm i.d. × 0.25 µm film thickness); acquisition scan range of 30–350 *m*/*z*. E-nose: 10 metal oxide sensors (MOS).	2023 [72]
17	In-tube dynamic extraction for analysis of volatile organic compounds in honey samples	Thirty-eight honey samples (Acacia, blossom, and forest)	ITEX-DHS	GC-MS	Tenax TA ITEX trap (polydiphenylene oxide); Optima FFAPplus fused-silica capillary column (60 m × 0.32 mm I.D., 0.5 μm film thickness); acquisition SCAN mode, *m*/*z* 40–200; acquisition SIM mode.	2022 [65]
18	Optimization of a miniaturized solid-phase microextraction method followed by gas chromatography mass spectrometry for the determination of twenty four volatile and semivolatile compounds in honey from Galicia (NW Spain) and foreign countries	Different types of honey samples including several varieties from Spain and some from Italy, France, Greece, and Kazakhstan.	HS-mini-SPME	GC-MS	Fiber: DVB/CAR/PDMS; DB-WAX capillary column (50 m × 0.20 mm i.d., 0.20 μm film thickness); acquisition SCAN mode, *m*/*z* 35–400.	2021 [61]
19	Volatile compounds in off-odor honey	Ten samples of off-odor honeys from Brazil	HS-SPME	GC-MS	Fiber: polydimethylsiloxane/divinylbenzene; VF-Wax MS capillary column (30 m × 0.25 mm internal diameter × 0.25 μm).	2021 [89]
20	Chemometrics exploration of monosaccharides, sugar acids, stable carbon isotopes, and volatile organic compounds in Malaysian stingless bee honey from different geographical origins	Fifteen multifloral stingless bee honey samples from Peninsular Malaysia	Liquid–liquid extraction	GC-MS	HP-5MS ((5%-phenyl)-methylpolysiloxane phase) (30 m × 0.25 mm × 0.25 μm film thickness); acquisition SCAN mode, *m*/*z* 40–650.	2024 [90]
21	*Rhamnus frangula* honey: screening of volatile organic compounds and their composition after short-term heating	Four *Rhamnus frangula* L. honey samples from North Croatia	HS-SPME and Ultrasonic Solvent Extraction (USE)	GC-MS and GC-FID	Fiber: divinylbenzene/Carboxen/polydimethylsiloxane (DVB/CAR/PDMS); pentane–Et2O (1:2, *v*/*v*) solvent used in USE; column: HP-5MS (30 m × 0.25 mm i.d., coating thickness: 0.25 μm); acquisition SCAN mode, *m*/*z* 30–300.	2015 [54]
22	Analysis of Volatile Compounds of Some Turkish Flower Honey Samples by Solid-Phase Microextraction and Gas Chromatography-Mass Spectrometry	Five different flower honeys from Turkey	HS-SPME	GC-MS	Fiber: PDMS/DVB; column: RTX-5MS (30 m × 0.25 mm × 0.25 µm).	2020 [50]
23	Quality of stingless bee honey based on volatile organic compounds and gas released	Stingless bee honey	-	FTIR coupled with White Gas Cell	Cyclone C5 gas cell with 2 to 8 m adjustable path length connected to the Frontier FTIR spectrometer with a deuterated triglycine sulphate (DTGS); range of spectrum set from 4000 cm^−1^ to 500 cm^−1^.	2020 [71]
24	Identification and Quantification of Volatile Compounds Released by Stingless Honey using Long Optical Path Infrared spectroscopy	Stingless bee honey from Malaysia	-	FTIR coupled with White Gas Cell	Cyclone C5 gas cell with 2 to 8 m adjustable path length connected to the Frontier FTIR spectrometer with a deuterated triglycine sulphate (DTGS); range of spectrum set from 4000 cm^−1^ to 450 cm^−1^.	2016 [70]
25	The Tracing of VOC Composition of Acacia Honey During Ripening Stages by Comprehensive Two-Dimensional Gas Chromatography	All honey samples and the related comb wax samples were from Slovakia	HS-SPME	GCxGC/TOF-MS	Fiber: DVB/CAR/PDMS; DB-FFAP column (30 m × 0.25 mm 0.25 µm) in the first dimension and BPX-50 column (1.5 m × 0.1 mm × 0.1 µm) in the second dimension; the signal acquisition rate was 100 spectra/s in the *m*/*z* range 29–450.	2016 [69]
26	The effect of low-temperature spray drying with dehumidified air on phenolic compounds, antioxidant activity, and aroma compounds of rapeseed honey powders	Rapeseed honey from a local apiarist in Poland	HS-SPME	GC-MS	Fiber: divinylbenzene/carboxene/polydimtheylsiloxane; ZB WAX plus capillary column (30 m × 0.25 mm × 0.25 μm); acquisition SCAN mode, *m*/*z* 35–500.	2019 [91]
27	The phenolic composition, aroma compounds, physicochemical and antimicrobial properties of *Nigella sativa* L. (black cumin) honey	Black cumin honey samples were obtained from experienced beekeepers in Turkey	HS-SPME	GC-MS	Fiber: divinylbenzene/carboxin/polydimethylsiloxane; 30 m 5 Ms column.	2023 [92]
28	Changes of various quality characteristics and aroma compounds of astragalus honey obtained from different altitudes of Adana-Turkey	Honey samples were obtained from honey producers as centrifugal honey in Turkey	Liquid–liquid extraction	GS-MS-FID	Extraction with dichloromethane and concentration of organic phase with Vigreux column; DB-WAX capillary column (30 m × 0.25 mm × 0.25 μm); acquisition SCAN mode, *m*/*z* 29–350.	2021 [59]
29	Untargeted and Targeted Discrimination of Honey Collected by *Apis cerana* and *Apis mellifera* Based on Volatiles Using HS-GC-IMS and HS-SPME-GC−MS	Several honey samples from *A. cerana* and *A. mellifera* collected in China	HS and HS-SPME	GC-IMS and GC-MS	FS-SE-54-CB-0.5 (15 m × 0.53 mm ID) column; drift tube operated at a constant voltage of 400 V/cm at 45 °C for GC-IMS. Fiber: divinylbenzene/carboxen/polydimethylsiloxane; capillary column: HP-DB-5 (30 m × 0.25 m × 0.25 μm); acquisition SCAN mode, *m*/*z* 15–200 for GC-MS.	2019 [67]
30	Chemical markers of a rare honey from the traditional spice plant *Amomum tsao-ko* Crevost et Lemari’e, via integrated GC–MS and LC-MS approaches	Linden, chaste, acacia, loquat, cherry, and rape honey samples were obtained from cooperative apiaries in China	HS-SPME	GC-MS	Fiber: DVB/CAR/PDMS; HP-5 capillary column (30 m × 0.25 mm × 0.25 μm).	2023 [93]
31	Fingerprinting chemical markers in the Mediterranean orange blossom honey: UHPLC-HRMS metabolomics study Integrating melissopalynological analysis, GC-MS and HPLC-PDA-ESI/MS	Some honey samples (*n* = 28) were collected in Italy, Greece, and Egypt, and another 12 citrus honey samples were purchased from an Italian market	HS-SPME	GC-MS	Fiber: polyacrylate; MEGA 5-HT column (30 m × 0.25 mm, 0.25 µm film thickness); acquisition SCAN mode, *m*/*z* 50–500.	2023 [51]
32	Optimization of the extraction of the volatile fraction from honey samples by SPME-GC-MS, experimental design, and multivariate target functions	Commercial organic multiflower honey	HS-SPME	GC-MS	Fiber: DVB/CAR/PDMS; capillary column: VF-5MS (30 m × 0.25 mm × 0.25 µm); acquisition SCAN mode, *m*/*z* 35–400.	2017 [94]
33	Authentication of chaste honey adulterated with high fructose corn syrup by HS-SPME-GC-MS coupled with chemometrics	Chaste honey samples (*n* = 15) were obtained directly from farmers at China bee farms	HS-SPME	GC-MS	Fiber: Polydimethylsiloxane/divinylbenzene; HP-5MS capillary column (60 m × 0.25 mm, 0.25 μm); acquisition SCAN mode, *m*/*z* 50–500.	2023 [53]
34	Screening of polish Fir honeydew honey using GC/MS, HPLC-DAD, and physical-chemical parameters: benzene derivatives and terpenes as chemical markers	Five samples of Fir honeydew honey were obtained from professional beekeepers in different parts of Poland	HS-SPME and ultrasound-assisted solvent extraction (USE)	GC-FID and GC-MS	Two fibers: PDMS/DVB and DVB/CAR/PDMS; two different solvents—a mixture of pentane with diethyl ether and dichloromethane were used for separate extractions (in case of USE); capillary column HP-5MS (30 × 0.25 mm, with coating thickness 0.25 μm); acquisition SCAN mode, *m*/*z* 30–300.	2017 [58]
35	Stir bar sorptive extraction coupled with GC/MS applied to honey: optimization of method and comparative study with headspace extraction techniques	A monofloral sample of eucalyptus honey (*Eucalyptus camaldulensis* Dehnh.) was obtained from a professional beekeeper from Italy	Stir bar sorptive extraction (SBSE)	GC-MS	Twister^®^ stir bar; VF-Wax capillary column (60 m × 0.25 mm i.d., 0.5 μm film thickness) and HP-5MS capillary column (30 m × 0.25 mm, film thickness 0.17 μm).	2017 [62]
36	Influence of beeswax adulteration with paraffin on the composition and quality of honey determined by physico-chemical analyses, 1H NMR, FTIR-ATR and HS-SPME/GC–MS	Honey ripened in honeycomb built on paraffin-based (90%) comb foundations (PF-H), and honey ripened in honeycomb constructed on comb foundations made of genuine beeswax (BWF-H)	HS-SPME	GC-MS	Fiber: DVB/CARB/PDMS; HP-5MS capillary column (5% phenylmethylpolysiloxane); acquisition SCAN mode, *m*/*z* 30–300.	2019 [95]
37	Unifloral Autumn Heather Honey from Indigenous Greek *Erica manipuliflora* Salisb.: SPME/GC-MS Characterization of the Volatile Fraction and Optimization of the Isolation Parameters	Twenty-five honey samples provided directly by Greek beekeepers	HS-SPME	GC-MS	Fiber: DVB/CAR/PDMS; Rtx-5MS (30 m × 0.25 mm i.d., 0.25 µm film thickness) chromatography column; acquisition SCAN mode, *m*/*z* 30–650.	2021 [96]
38	GC–MS investigations of VOCs in South Indian honey samples as environmental biomarkers	Twenty-five honey samples collected from the Western Ghats, India	Liquid–liquid extraction	GC-MS	Extraction with dichloromethane and injection; Rtix 5 MS colum; acquisition SCAN mode, *m*/*z* 50–800.	2021 [57]
39	The Microscopic and GC-MS Analysis of Turkish Honeydew (Pine) Honey	Seventy-eight honeydew (Pine) honey samples were collected from ten areas of Turkey	Liquid–liquid extraction	GC-MS	Extraction with methanol and then with ethanol. After sample drying, this was dissolved in 0.5 mL of ethanol; DB 5MS column (30 m × 0.25 mm and 0.25 μm of film thickness).	2016 [97]
40	HS-SPME-GC-MS Analysis of the Volatile Composition of Italian Honey for Its Characterization and Authentication Using the Genetic Algorithm	Ninety-eight honey samples from Italian honey producers, with the exception of four Greek samples	HS-SPME	GC-MS	Fiber: DVB/CAR/PDMS; DB InnoWAX column (0.4 μm × 0.2 mm × 50 m); acquisition SCAN mode, *m*/*z* 29–350.	2024 [98]
41	Comparison of original honey (*Apis* sp. and *Tetragonula *sp.) and fake honey compounds in Indonesia using gas chromatography-mass spectrometry (GC-MS)	Honeys from *Apis *sp. (*Apis melifera*, *A. dorsata*, *A. cerana*) and *Tetragonula *sp. from several regions in Indonesia. Fake honey samples (*n* = 20) were prepared by adding fructose, NaHCO3, and Aquadest	Liquid–liquid extraction	GC-MS	No details are reported.	2019 [99]
42	HS-SPME/GC-MS metabolomic analysis for the identification of exogenous volatile metabolites of monofloral honey and quality control suggestions	Total of 203 monofloral honey samples of different botanical origins (citrus, fir, pine, and thyme)	HS-SPME	GC-MS	Fiber: divinyl benzene/carboxen/polydimethylsiloxane (DVB/CAR/PDMS); DB-5MS column (cross-linked 5% PH ME siloxane) (60 m × 320 µm i.d., × 1 µm film thickness); acquisition SCAN mode, *m*/*z* 50–550.	2022 [100]
43	Characterization of Summer Savory (*Satureja hortensis* L.) Honey by Physico-Chemical Parameters and Chromatographic/Spectroscopic Techniques (GC-FID/MS, HPLC-DAD, UV/VIS and FTIR-ATR)	Sample of *Satureja hortensis* L. honey was obtained from a plantation in Poland	HS-SPME and Ultrasonic Solvent Extraction	GC-MS and GC-FID	Fiber: divinylbenzene/carboxene/polydimethylsiloxane (DVB/CAR/PDMS); extraction with pentane:Et2O 1:2 (*v*/*v*) mixture and concentration of the organic phase with distillation with Danish–Kuderna apparatus; the same column was used for GC-MS and FID: 30 m capillary column HP-5MS (5% phenyl-methylpolysiloxane); acquisition SCAN mode, *m*/*z* 30–300 for GC-MS.	2015 [56]
44	GC-MS Analysis of the Volatile Constituents and Antioxidant Activity of the Crude Honey Residue from Takum Local Government Area of Taraba State, Nigeria	The crude honey was collected from the wild in Takum Local Government Area of Taraba State, Nigeria	Hydrodistillation	GC-MS	The extraction lasted for two (2) hours and the extract was collected over hexane; column: HP-5MS (30 m × 0.320 mm; 0.25 μm thickness); acquisition SCAN mode, *m*/*z* 35–500.	2022 [63]
45	Screening of *Satureja subspicata* Vis. Honey by HPLC-DAD, GC-FID/MS and UV/VIS: Prephenate Derivatives as Biomarkers	Ten samples of *Satureja subspicata* Vis. honey were obtained from professional bee-keepers in Croatia (the bee species was *Apis mellifera carnica*)	Ultrasonic Solvent Extraction	GC-MS and GC-FID	Two solvents were used for extraction: pentane:Et2O 1:2 (*v*/*v*) mixture and CH2Cl2. Sample was concentrated with distillation with Danish–Kuderna apparatus; the same column was used for GC-MS and FID: capillary column: HP-5MS (5% phenyl-methylpolysiloxane) (30 m × 0.25 mm; 0.25 µm); acquisition SCAN mode, *m*/*z* 29–350 for GC-MS.	2016 [55]

## 3. Volatile Organic Compounds in Honey: Types and Geographical Origin

The analysis of volatile organic compounds (VOCs) across different honey samples reveals a diverse chemical composition influenced by floral sources and geographical origin. Aromas are distinguished not only by the type of bee from which the product is collected but also by complex factors such as the floral source (monofloral or multifloral), the environmental conditions, and geographical place. This distinction in aroma certainly highlights the complexity of volatile compound groups. To characterize the uniqueness of honey types, most studies investigate the compound groups with the highest concentration. For instance, a study [10] identified distinct VOC profiles that contribute to the unique aroma characteristics of honey from four different ecoregions of Argentina. By analyzing 25 honey samples from the Pampa, Paraná Delta, Espinal, and Patagonian Forest regions, the research underscores the significant role of local flora and environmental conditions in shaping the sensory and chemical properties of honey. The variation in VOC profiles among different honey types demonstrates the strong influence of floral origin, environmental conditions, and processing factors on honey aroma. Paraná Delta Honey (eucalyptus honey) exhibited high concentrations of cineole, pinene, limonene, and furfural, imparting a medicinal, herbal, and slightly citrusy aroma. The predominance of cineole, pinene, and limonene in Paraná Delta Honey aligns with previous studies [1,26,57,80,101,102] showing that eucalyptus-derived honey is rich in monoterpenes, which contribute to its characteristic medicinal and herbal scent. These compounds are also known for their antimicrobial properties, suggesting potential functional benefits beyond flavor [103,104,105].

In contrast, the aldehydes and ketones found in Espinal Honey (Sunflower Honey) reflect volatile compounds derived from lipid oxidation and Maillard reactions [7,25]. Hexanal is a key marker for green and nutty aromas in honey, while octanone contributes with subtle fruity notes. The presence of methyl esters in this sample suggests additional enzymatic modifications occurring during nectar transformation [39,106].

Pampa Honey exhibited a more balanced composition, with phenylethyl alcohol and benzaldehyde contributing to its delicate floral aroma. The detection of hydroxymethylfurfural (HMF), a thermal degradation product of sugars, suggests possible heat exposure or natural aging. While HMF is considered a quality indicator in honey [101,107], its presence within regulatory limits does not necessarily affect sensory quality but may provide information about storage conditions.

In addition, Patagonian Honey (Native Forest Honey) displayed a more complex VOC composition, including terpenes (limonene, pinene), phenylethyl alcohol, and furans, producing a complex floral and earthy aroma. The occurrence of furans suggests either thermal processing or prolonged storage, as these compounds can form due to carbohydrate degradation [7,14,108]. The presence of phenylethyl alcohol reinforces the floral complexity of this honey type, making it a potentially valuable product for gourmet applications [14,29,108].

### 3.1. Floral Origin

The floral origin of honey plays a key role in the characteristics of honey quality evaluation. The study by Baroni [101] offers a significant contribution to the field of honey authentication by investigating the volatile organic compound (VOC) profiles of five monofloral honey types (alfalfa, sunflower, white clover, carob, and calden) from Argentina. By analyzing 42 monofloral honey samples using HS-SPME coupled with GC-MS, the researchers identified and quantified 35 VOCs, which served as botanical fingerprints for distinguishing honey based on floral origin [101]. The application of chemometric techniques, including hierarchical cluster analysis, stepwise discriminant analysis, and the k-nearest-neighbor discriminant method, was particularly noteworthy. These methods effectively reduced the complexity of the VOC data to six key compounds—octanal, benzene acetaldehyde, 1-octanol, 2-methoxyphenol, nonanal, and 2-H-1-benzopyran-2-one—achieving a 93% correct classification rate. This highlights the potential of chemometrics in simplifying and enhancing the accuracy of honey classification based on VOC patterns [101,106].

Their research revealed that while these key VOCs varied quantitatively across different floral origins, none were exclusive to a single honey type. This suggests that the unique aroma and chemical profile of each monofloral honey arise from a combination of VOCs rather than individual markers. The findings underscore the complexity of honey’s chemical composition and the importance of considering multiple compounds when evaluating authenticity and quality. Furthermore, the research demonstrates the utility of VOC profiling as a reliable tool for differentiating monofloral honeys, which is crucial for both producers and consumers in ensuring product authenticity. By linking specific VOCs to floral origins, the study also provides a foundation for further research into the relationship between botanical sources and honey’s sensory properties [41,50,51,109,110,111,112].

### 3.2. Geographical Origin

The geographical origin of honey significantly influences its volatile organic compound (VOC) composition, infusing distinct sensory and chemical characteristics that reflect regional floral sources and environmental conditions (Table 2). For instance, honey from Argentina exhibits a rich diversity of terpenes, esters, and phenolic compounds, contributing to its complex floral and herbal aroma profile. This is likely due to the diverse native flora, such as clover and eucalyptus [10,35]. In contrast, Chinese honey is characterized by elevated levels of aldehydes and acetate derivatives, yielding fruity and floral notes, which is possibly linked to dominant floral sources like lychee and longan [44,46,113]. Indian honey stands out with its unique sulfurous, spicy, and medicinal attributes, driven by VOCs such as azadirachtin and zingiberene, which may originate from neem and ginger-influenced nectar [25,62,114]. European honeys—particularly those from Portugal, Poland, Greece, and Spain—are distinguished by high concentrations of benzaldehyde and linalool derivatives, contributing to citrusy, nutty, and sweet aromatic profiles, often associated with lavender, thyme, and heather blossoms [5,24,26,39,56,115]. These regional variations not only highlight the profound impact of terroir on honey chemistry but also underscore the potential of VOC fingerprinting as a reliable tool for geographical authentication. Furthermore, understanding these differences can aid in quality assessment, fraud prevention, and the preservation of regional honey typicity in global markets.

Certain honey varieties exhibit remarkably distinctive aroma profiles due to the presence of rare or region-specific volatile organic compounds (VOCs), offering unique sensory experiences and potential bioactive properties. For example, Neem honey from India contains azadirachtin, a bioactive compound that gives a pronounced medicinal and slightly bitter character, likely derived from the nectar of Azadirachta indica flowers, which are traditionally used in Ayurvedic medicine [25,114]. Similarly, Jujube honey, prevalent in China and Sudan, is characterized by high levels of uridine and proline-derived VOCs, which contribute to its distinctive fruity sweetness with a subtle tanginess, reflecting the influence of Ziziphus jujuba blossoms [14,44,57]. In contrast, Strawberry Tree honey from Portugal contains α-isophorone and trimethylphenol, yielding a complex bitter, earthy, and caramel-like aroma, which is highly prized despite its acquired taste [24]. Meanwhile, honeydew honey from Poland, primarily derived from aphid secretions rather than floral nectar, contains valeric acid methyl ester, lending it woody and fermented notes that distinguish it from traditional floral honeys [5,107,108,109,110,111]. These unique VOC signatures not only enhance the gastronomic value of these specialty honeys, but also hold potential for authentication, fraud prevention, and even nutraceutical applications, due to their bioactive constituents.

### 3.3. Entomological Origin

Beyond honey from Apies mellifera (honeybee), the VOCs of stingless bee honey are also examined. Stingless bee honey is more often expensive than honey from Apis mellifera because of its limited production, perceived medicinal value, and the traditional methods of harvesting, which can damage nests and reduce yields [7]. In this review, VOC analyses in honey produced by four species of stingless bees and comparison with honey from Apis mellifera (honeybee) are done using HS-SPME -GC-MS: the research identifies 284 VOCs, highlighting unique chemical profiles for each honey type. Research results show that only eight VOCs were common across all six honey samples: ethanol, acetic acid, isopentanol, 2-methylbutan-1-ol, benzaldehyde, limonene, 2-ethylhexan-1-ol, and 2-phenylethanol [7,19,111,112].

In general, the stingless bee honeys showed significantly higher concentrations of aliphatic acids and esters, contributing to their unique aroma profiles. Safranal was notably high in Scaptotrigona postica honey, giving it a distinctive saffron-like aroma. Also, Tetragona clavipes honey contained abundant octanoic acid and sesquiterpenes, while Melipona quadrifasciata honey had high levels of aromatic compounds (notably ethyl benzoate and 2-phenylethanol) [27,35].

Apis mellifera honeys exhibited a higher diversity of monoterpenoids and furans (e.g., furfural), differentiating themselves from stingless bee honeys. The study suggests VOCs as effective markers for identifying honey’s botanical and entomological origins, aiding in quality control and authenticity verification [7,27,113].

Stingless bee honeys, such as those produced by the Mandaguarí Negra, Borá, Yateí, and Uruçu species in Argentina and Brazil, exhibit remarkably distinct volatile organic compound (VOC) profiles compared to traditional Apis mellifera honeys, reflecting their unique floral sources and biochemical processing by meliponine bees. These honeys are characterized by high concentrations of acetic acid and safranal, which contribute to their pronounced sour, saffron-like, and herbal aromatic notes—a sensory profile rarely found in conventional honeys [10,35]. Additionally, their richness in terpenoids and esters infuses complex floral, fruity, and balsamic nuances, likely derived from the diverse tropical and subtropical flora these bees forage on, including native shrubs and resin-producing plants. The elevated presence of such compounds suggests that stingless bee honeys undergo different enzymatic and fermentation processes during storage in their resinous pots, further differentiating their chemical signatures. These unique VOC profiles not only enhance their culinary appeal but also present opportunities for geographical and entomological authentication, protecting against mislabeling in niche markets. Moreover, the bioactive potential of terpenoids and safranal deserves further investigation into their anti-inflammatory, antioxidant, and antimicrobial properties [7,8].

**Table 2:** Aroma profile of honey: volatile organic compounds from different honey samples.

Name of the Honey	Botanical Origin (Nectar Source)	Terpenes	Aldehydes	Ketones	Other VOCs	Aroma Profile	Geographical Origin
Paraná Delta *	*Eucalyptus* spp.	Cineole; a-pinene; limonene	Furfural	-	-	Medicinal, herbal, slightly citrusy	Paraná Delta and Islands, Argentina [10]
Espinal *	*Helianthus annus*	-	Hexanal	Octanone	Methyl esters	Fruity, floral, slightly nutty	Espinal, Argentina [10]
Pampa **	*Trifolium* spp.	Limonene	Benzaldehyde	-	Hydroxymethyl furfural (HMF); Phenylethyl alcohol	Sweet, floral, mild buttery undertones	Pampa, Argentina [10]
Patagonian **	*Nothofagus*, *Berberis*	Limonene; Pinene	-	-	Phenylethyl alcohol; Furans	Complex, floral, earthy	Patagonian Forest, Argentina [10]
Alfalfa *	*Medicago sativa*	-	Benzenea- cetaldehyde; Nonanal	-	2-methoxyphenol	Sweet, floral, slight citrusy undertones	Córdoba, Argentina [32]
Sunflower *	*Helianthus annuus*	-	octanal	-	2-Methoxyphenol	Earthy, herbal with low sweetness	Córdoba, Argentina [32]
White Clover *	*Melilotus albus*	-	Benzene acetaldehyde	2-H-1-Benzopyran-2-one	-	Mildly sweet, vanilla-like aroma	Córdoba, Argentina [32]
Carob *	*Prosopis* spp.	-	Nonanal; Octanal	-	-	Woody, slightly nutty, and floral	Córdoba, Argentina [32]
Caldén *	*Prosopis caldenia*	-	Nonanal	-	1-Octanol	Fruity, with faint herbal notes	Córdoba, Argentina [32]
Apies cerana *	*Litchi chinensis*	-	-	-	1-Nonanol; 1-Heptanol; Phenethyl Acetate	Sweet, fruity (pineapple, grape), herbaceous	Guangdong, Gansu, Shaanxi, China [26]
Apies mellifera *	*Litchi chinensis*	-	Benzaldehyde; Heptanal; Phenylacetaldehyde	-	-	Floral (hawthorne, lavender), almond, citrus	Guangdong, Henan, Sichuan, China [26]
Citrus (Egypt) *	*Citrus sinensis*	Linalool	2-Methylbutanal; Benzaldehyde	Heptane	Methyl anthranilate;	Fruity, floral, citrusy notes	Egypt [36]
Citrus (Greece) *	*Citrus reticulata*	p-cymene	Nonanal; Decanal; Lilac Aldehydes	-	-	Sweet, floral, and slightly spicy	Greece [36]
Citrus (Morocco) *	*Citrus aurantium*	-	Benzaldehyde	-	Dill Ether; Cis-Linalool Oxide	Herbal, earthy, slightly fruity	Morocco [36]
Citrus (Spain) *	*Citrus limon*	-	-	-	Ethyl octanoate; ethyl nonanoate; ethyl acetate	Mild floral, light fruity, and citrusy	Spain [36]
Winter *	*Schefflera actinophylla*	-	Benzaldehyde; Phenylacetaldehyde; Heptanal	2-heptanone	-	Sweet, floral, slightly spicy	Southern China [106]
Sapium *	*Triadica sebifera*	-	Lilac aldehydes	-	Phenylethyl acetate; hotrienol	Lightly sour, rough texture, low concentration	Southern China [106]
Litchi *	*Litchi chinensis*	Cis-rose oxide; Trans-linalool oxide	Lilac aldehydes	-	-	Floral, fruity, sweet	India (Karnataka) [22]
Neem *	*Azadirachta indica*	Azadirachtin; Germacrene	-	-	Dipropyl disulphide	Medicinal, slightly bitter, sulfurous	India (Karnataka) [22]
Ginger *	*Zingiber officinale*	Zingiberene; Farnesene	Octadecenal			Spicy, woody, warm	India (Karnataka) [22]
Eucalyptus *	*Eucalyptus* spp.	-	Phenylacetaldehyde	Acetoin	2-Hydroxycineole	Herbal, woody, slightly minty	India (Karnataka) [22]
Lemon *	*Citrus limon*	Limonene diol	-	-	Limonyl alcohol; Methyl anthranilate	Sweet, citrusy, floral	India (Karnataka) [22]
Kashmiri White **	*Trifolium repens*	-	-	Furyl hydroxymethyl ketone	Heptanol	Mild, light floral	India (Kashmir) [22]
B.R. Hill **	*Emblica officinalis*	Cis-linalool oxide	2-Pentyl-2-nonenal	-	Caffeine	Earthy, robust, slightly woody	India (Karnataka) [22]
Pan India *	*Brassica campestris*/spp.	-	-	-	Ethyl oleate; Decane; Nonadecanol	Mild, mixed floral	India (Nationwide) [22]
Lycium *	*Lycium barbarum*	-	-	-	β-Glucose; Melezitose; Xylobiose	Sweet, mildly floral	Ningxia, China [42]
Jujube *	*Ziziphus jujuba* Mill.	-	-	-	Proline; Uridine;	Fruity, rich, slightly tangy	Henan, China [42]
Linden *	*Tilia* spp.	-	-	-	Melezitose; Lysine	Herbal, woody, floral	Inner Mongolia, China [42]
Locust *	*Robinia pseudoacacia* L.	-	-	-	Xylobiose; Melezitose	Light, delicately floral	Jiangsu, China [42]
Sunflower *	*Helianthus annuus*	-	-	-	Proline; β-Glucose	Nutty, earthy, floral	Xinjiang, China [42]
Multifloral **	*Prunus padus*	-	-	-	Proline; Melezitose	Mixed floral, complex	Sichuan, China [42]
Chaste *	*Vitex negundo*	-	-	-	Xylobiose; Lysine	Sweet, herbal, slightly spicy	Hubei, China [42]
Eriobotrya *	*Eriobotrya japonica*	-	-	-	Uridine, Turanose	Mild fruity, subtly floral	Guangdong, China [42]
Mandaguarí Negra ***	*Scaptotrigona postica*	Linalool	Safranal	-	Hotrienol oxide; Acetic acid	Floral, saffron-like, fruity	Misiones, Argentina [9]
Borá ***	*Tetragona clavipes*	β-caryophyllene; sesquiterpenes	-	-	Octanoic acid; Ethyl octanoate	Fruity, fatty, slightly spicy	Misiones, Argentina [9]
Mandazaia ***	*Melipona quadrifasciata quadrifasciata*	-	-	-	Ethyl benzoate; Diethyl succinate; 2-phenylethanol	Sweet, fruity, floral	Misiones, Argentina [9]
Yateí ***	*Tetragonisca fiebrigi*	-	-	β-damascenone	Acetic acid; Benzyl alcohol	Herbal, fruity, lightly floral	Misiones, Argentina [9]
Honeybee (Sample 5) **	*Trifolium* spp.	Linalool; monoterpenoids	Lilac aldehydes; Furfural	-	-	Sweet, floral, caramel-like	Wanda, Misiones, Argentina [9]
Honeybee (Sample 6) **	*Trifolium* spp.	β-pinene; limonene	Furfural; Lilac aldehydes			Floral, woody, caramel notes	Eldorado, Misiones, Argentina [9]
Rape **	*Brassica napus*	-	Benzaldehyde; 2-methyl-butanal	-	Acetone; Hotrienol	Mild, floral, slightly nutty	West Pomeranian, Poland [4]
Lime **	*Tilia* spp.	Dehydro-p-cymene	-	-	Menthofuran; Terpinen-4-ol	Herbal, citrusy, woody	Podlaskie, Poland [4]
Meadow & Marsh **	*Trifolium* spp.	-	Grandlure IV		1,4-butanediol diacetate; p-cymen-8-ol	Floral, fruity, smoky	Podlaskie, Poland [4]
Lime *	*Tilia* spp.	-	Grandlure IV; Furfural	-	Terpinen-4-ol	Lightly herbal, floral	Poland [4]
Buckwheat *	*Fagopyrum esculentum*	-	Furfural; Phenylacetaldehyde	-	Isovaleric acid; Ethanol	Strong, malty, earthy, pungent	Poland [4]
Honeydew *	*Abies* spp.	-	-	Acetoin	Valeric acid methyl ester; Isoamyl alcohol	Woody, resinous, fermented notes	Poland [4]
Arabian Jujube (H1) *	*Ziziphus spina-christi*	-	Furfural; Benzaldehyde	-	Phenylethyl alcohol	Sweet, fruity, floral	Ad. Damazin, Sudan [11]
Scented Thorn (H2) *	*Acacia nilotica*	-	2-Methylbutanal; Safranal	-	Benzyl alcohol	Floral, woody, slightly spicy	Ad. Damazin, Sudan [11]
Talh (H3) *	*Vachellia seyal*	-	Furfural; Phenylacetaldehyde	-	1-butanol	Earthy, resinous, caramel-like	Ad. Damazin, Sudan [11]
Multifloral (H4) **	*Multifloral sources*	-	Benzaldehyde; Hexanal	-	1-pentanol	Mild, mixed floral	Kabam, Sudan [11]
Multifloral (H5) **	*Multifloral sources*	-	Furfural	-	Phenylethyl alcohol; Methyl salicylate	Fruity, herbal	Um Dafoug, Sudan [11]
Arabian Jujube (H6) *	*Ziziphus spina-christi*	-	Safranal	-	Benzyl alcohol; Phenylethyl alcohol	Rich, fruity, floral	Wadisaleh, Sudan [11]
Multifloral (H7) **	*Multifloral sources*	-	2-methylbutanal; Furfural	-	Phenol derivatives	Floral, herbal, woody	El Obeid, Sudan [11]
Lisan Tair (H8) *	*Amaranthus graecizans*	-	Benzaldehyde; Furfural	-	Phenylethyl alcohol	Sweet, floral, slightly nutty	El Obeid, Sudan [11]
Talh (H9) *	*Vachellia seyal*	-	Safranal	-	Benzyl alcohol; Butanoic acid	Woody, herbal, earthy	Al Qadarif, Sudan [11]
Ban (H10) *	*Eucalyptus* spp.	-	Benzaldehyde; furfural	-	Phenylethyl alcohol	Fruity, floral, fresh	Al Qadarif, Sudan [11]
Acacia (Zone 1) *	*Robinia pseudoacacia*	Linalool oxide	Benzaldehyde	-	3-methyl-3-buten-1-ol; Acetic acid	Floral, sweet, mild fruity	Transylvania, Romania [108]
Acacia (Zone 2) *	*Robinia pseudoacacia*	-	-	5-ethenyldihydro-5-furanone	Ethanol; Acetic acid; Benzyl alcohol	Fruity, acidic, fresh	Southern Romania [108]
Acacia (Zone 3) *	*Robinia pseudoacacia*	trans-Linalool oxide	Benzeneacetaldehyde; Hotrienol	Acetone	-	Floral, slightly woody, aromatic	Eastern Romania [108]
Carob Tree *	*Ceratonia siliqua*	α-Pinene; Linalool oxide	-	-	Methyl anthranilate	Woody, slightly floral	Algarve, Portugal [21]
Chestnut *	*Castanea sativa*	α-pinene	Benzaldehyde	Acetophenone	-	Nutty, strong, slightly bitter	Trás-os-Montes, Portugal [21]
Eucalyptus *	*Eucalyptus* spp.	α-Pinene	-	-	Aromadendrene; Eudesmol	Herbal, woody, slightly minty	Beira Baixa, Portugal [21]
Bell Heather *	*Erica* spp.	Linalool oxide	Benzene acetaldehyde; Hotrienol	-	-	Floral, spicy, resinous	Trás-os-Montes, Portugal [21]
Incense *	*Pittosporum undulatum*	Limonene	-	-	Benzyl salicylate; α-terpineol	Woody, balsamic, slightly floral	Azores, Portugal [21]
Lavender *	*Lavandula* spp.	-	n-Nonanal; Decanal	-	Benzyl alcohol	Sweet, aromatic, floral	Beira Baixa, Portugal [21]
Orange *	*Citrus* spp.	-	Lilac aldehydes	-	Methyl anthranilate; indole	Citrus, fruity, floral	Algarve, Portugal [21]
Rape *	*Brassica napus*	-	-	-	Dimethyl trisulfide	Mild, slightly sulfurous	Alentejo, Portugal [21]
Raspberry *	*Rubus idaeus*	Linalool oxide	Hotrienol; lilac aldehydes	-	-	Fruity, floral, fresh	Estremadura, Portugal [21]
Rosemary *	*Rosmarinus officinalis*	Linalool oxide	Benzaldehyde; Hotrienol	-	-	Herbal, floral, slightly minty	Algarve, Portugal [21]
Sunflower *	*Helianthus annuus*	α-Pinene; β-copaene	Benzene acetaldehyde			Earthy, nutty, slightly woody	Baixo Alentejo, Portugal [21]
Strawberry Tree *	*Arbutus unedo*	-	-	α-Isophorone; 4-keto-isophorone	Trimethylphenol	Bitter, earthy, caramel-like	Algarve, Portugal [21]
Guairapó ***	*Multifloral sources*	Linalool oxide	Hotrienol; benzaldehyde	-	-	Floral, fruity, slightly herbal	Guaraqueçaba, Cambará, Brazil [24]
Mandurí ***	*Multifloral sources*	Linalool oxide	--		Benzyl alcohol; Epoxylinalool	Floral, light citrusy	Guaraqueçaba, Cambará, Brazil [24]
Uruçu ***	*Multifloral sources*	Linalool	Benzaldehyde; Hotrienol	-	-	Sweet, floral, mild woody	Guaraqueçaba, Cambará, Brazil [24]
Mandaçaia ***	*Multifloral sources*	-	Hotrienol,	-	Benzyl alcohol; Ethyl benzoate	Herbal, slightly fruity	Guaraqueçaba, Cambará, Brazil [24]
Borá ***	*Multifloral sources*	-	Benzaldehyde	-	Ethyl octanoate; Ethyl decanoate	Strong, fruity, slightly acidic	Guaraqueçaba, Cambará, Brazil [24]
Jataí ***	*Multifloral sources*	Linalool	Lilac aldehydes	-	benzyl alcohol	Floral, light caramel	Guaraqueçaba, Cambará, Brazil [24]
Tubuna ***	*Multifloral sources*	Linalool oxide	-	2-Heptanone	2-Heptanol	Fruity, resinous, slightly spicy	Guaraqueçaba, Prudentópolis, Brazil [24]
Tujumirim ***	*Multifloral sources*	Linalool oxide	Hotrienol	-	Benzyl salicylate	Woody, herbal, balsamic	Guaraqueçaba, Brazil [24]

* Monofloral honey, ** multifloral honey, *** stingless bee honey.

### 3.4. VOCs Formation Pathways

Honey’s VOCs form generally from plant nectar. During nectar-to-honey transformation, some phytochemicals pass through the bee’s stomach unchanged, while others change via bee enzymes, heat, processing and storage, or microbial and environmental factors [12]. The flavor of honey is not the result of a single process; rather, it develops through a multistage transformation pathway. The formation of honey flavor can be divided into the following processes: direct botanical inputs, enzymatic transformations during nectar processing by bees, and post-collection processes dominated by microbial fermentation and abiotic reactions. VOCs in honey generally originate from the nectar sources, which harbor plant-derived phytochemicals such as terpenes (e.g., linalool, hotrienol), benzene derivatives, aldehydes, and ketones that define the specific nectar aroma. Some pass unaltered through the bee’s honey stomach, evading full enzymatic breakdown during ripening [12,13]. However, since concentrations of volatiles and chemical forms are altered during the processing stages, these volatiles derived from plants could be considered precursors rather than final aroma determinants [116,117,118,119,120,121,122].

Enzymatic changes occur in the bee stomach through invertases. During these enzymatic transformations, sucrose hydrolyses into glucose and fructose, and glucose oxidase significantly increases the pool of fermentable sugars like gluconic acid and hydrogen peroxide, contributing to acidity and antimicrobial qualities as well as indirectly influencing flavor stability. These enzymatic reactions change the pH, reduce water activity, and create reactive intermediates that favor the release of bound aroma compounds (e.g., glycosidically bound terpenes) and encourage the growth of microorganisms [120,121,122]. These transformations, along with bee metabolism, heat from regurgitation, and storage handling, modify VOCs—producing new ones or degrading heat-labile compounds—while bees may control minor fermentation with symbiotic microbes [13,15].

Microbial fermentation has been a long-ignored factor in honey maturation, but recent metagenomic and fermentation studies show that specific yeast and bacterial communities transform honey constituents into VOCs, particularly during honey collection, storage, or dilution [121]. Hive microbiota, including osmotolerant yeasts (*Starmerella*, *Metschnikowia*, *Hanseniaspora*, *Zygosaccharomyces*) convert sugars and amino acid precursors into higher alcohols (isoamyl alcohol, 2-phenylethanol), esters (2-phenyl acetate), terpenoid derivatives (hotrienol), and organic acids (succinate) that directly improve sensory quality [121], whereas bacteria (*Gluconobacter*, *Lactobacillus*), contribute to VOCs through fermentation of nectar and bee bread. Yeasts produce secondary metabolites and volatiles that modulate aromas and suppress spoilers, while bacteria aid in acid production; this occurs in stored provisions like ripening honey. Fermentation enhances flavor complexity but is limited by honey’s low water activity and acidity [17,18,19]. As an example, acetic acid and ethanol rank among the primary volatiles in honeys from varied botanical and geographical sources. Acetic acid is generated mainly through bee metabolism during nectar processing and honey ripening, while ethanol comes largely from yeast-driven carbohydrate fermentation. These compounds’ levels depend on the floral origins, environment, moisture, and storage, which can spur microbial activity [20]. Beyond biotic processes, abiotic reactions also influence honey volatiles during storage and processing. Furans, aldehydes, and caramel-like notes are generated from Maillard reactions between reducing sugars and amino acids, particularly at high temperatures or over long storage durations; the oxidation of lipids and terpenes can produce aldehydes and ketones, and additional aroma-active compounds can be released by hydrolytic reactions, which are typically slower than microbial transformations, but which contribute to aged or thermally processed honey flavor profiles. During storage, a general decrease in organic acids is commonly observed, particularly for acetic and formic acids. Formic acid is naturally present in fresh honey due to its occurrence in plants visited by honeybees, and its rapid decline over time has also been reported in hive matrices following its application as an acaricide against *Varroa destructor*. Acetic acid, however, may show a less pronounced decrease during storage, as it can also be generated through mild fermentation processes, especially at elevated temperatures. For instance, at 35 °C, some acids—such as butenoic and 2-butenoic acids—exhibit greater temporal stability and higher relative abundance compared to lower storage temperatures. Given their association with rancid sensory notes, these compounds may serve as additional indicators of honey degradation under thermal stress. In contrast, monoterpenoids such as thymol, commonly used in apiculture as a miticidal agent, appear to be more persistent during storage than organic acids [121]. The best way to characterize the formation of honey flavor is a dynamic multilevel system wherein bee enzymes, microbial metabolism, and abiotic chemistry sequentially transform precursors derived from plants. A clear mechanistic roadmap and alignment of honey research with established paradigms in food fermentation and flavor chemistry could be provided by a schematic drawing that links primary botanical inputs to secondary enzymatic and microbial pathways, and ultimately to major VOC classes (terpenes, alcohols, esters, organic acids, and furans) [122,123,124,125,126].

### 3.5. VOC Profiles in Adulteration Detection

Honey adulteration remains a global concern that undermines consumer trust and the sustainability of the honey industry. Volatile organic compound (VOC) profiling has proven effective in discriminating honey according to botanical and geographical origin, as well as in detecting adulteration. When coupled with chemometric analysis, VOC-based approaches provide rapid, cost-effective, and robust screening tools for honey authenticity assessment and quality control. In general, reported adulteration practices can be categorized into direct and indirect forms. Direct adulteration includes the addition of sugar syrups, blending with lower-quality honeys, and mislabeling of botanical, geographical, or organic origins, whereas indirect adulteration involves practices such as artificial feeding of bee colonies, premature harvesting of unripe honey, and improper processing or storage [121]. As an example, a recent study has demonstrated a rapid method for coriander honey adulteration detection by combining HS-SPME–IMS with chemometric analysis. The approach differentiated authentic honey from samples adulterated with inverted sugar, HFCS, and sugar-fed honey, using VOC fingerprinting and machine learning models. Studies also highlight that certain VOC profile concentration levels can indicate honey adulteration, for instance, with octanal, nonanal, phenylacetaldehyde, and β-Damascenone [123,124,125,126,127].

## 4. Conclusions

The diversity of volatile organic compounds (VOCs) in honey underscores the profound impact of nectar sources and geographical origin on its unique aroma profile. This variability not only reflects the intricate relationship between bees, plants, and environmental factors but also presents a valuable opportunity for advancing honey authentication and quality control measures. VOC analysis emerges as a powerful tool for distinguishing authentic honey from adulterated products, ensuring transparency in the market while protecting consumer trust. The interpretation of honey VOC profiles depends on analytical tools, methods, and sample handling, with HS-SPME-GC/MS remaining the reference technique for volatile characterization. Despite significant advances, some gaps still remain, including the limited application of real-time VOC monitoring, combining analytical and sensor-based techniques for comprehensive honey fingerprinting. To fully harness this potential, future research should prioritize the exploring of standardized VOC profiles for reliable authenticity verification, application of real-time VOC monitoring, and identification of honey adulteration by VOCs, as well as explore the potential health benefits associated with specific bioactive VOCs. Additionally, investigating the effects of climate change on honey VOC compositions is crucial, as shifting environmental conditions may alter floral availability and, consequently, the sensory and functional properties of honey. By deepening our understanding of honey VOCs, we do not only enhance consumer appreciation of this natural product but also contribute significantly to the broader fields of food authenticity, nutritional science, and quality assurance. Ultimately, integrating harmonized VOC analysis with a multi-analytical approach combining robust chemical markers and advanced data analysis into regulatory frameworks and industrial practices will ensure the preservation of honey’s integrity while promoting innovation in apicultural and food sciences.

## Data Availability

No new data were created or analyzed in this study. Data sharing is not applicable to this article.

## Abbreviations

The following abbreviations are used in this manuscript.

VOC	Volatile organic compounds
GCMS	Gas chromatography and mass spectrometry
HS-GCMS	Headspace solid extraction, gas chromatography, and mass spectrometry
FTIR	Fourier Transform Infrared Spectroscopy
GC-MS/MS	Gas chromatography and tandem mass spectrometry
GC-QTOF	Gas Chromatography Quadrupole Time-of-Flight
GC-O	Gas chromatography–olfactometry
GC-IMS	Gas Chromatography–Ion Mobility Spectrometry
GC-FID	Gas Chromatography with Flame Ionization Detection
GCxGC-MS	Two-dimensional gas chromatography mass spectrometry

## References

[1] Moldakhmetova G., Kurmanov R., Toishimanov M., Tajiyev K. (2023). Palynological, Physicochemical, and Organo-leptic Analysis of Honey from Different Climate Zones of Kazakhstan. Casp. J. Environ. Sci..

[2] Maikanov B., Auteleyeva L., Satayeva Z., Aipova A. (2023). The Development of Balqymyz Beverage from Honey and Koumiss. J. Agric. Food Res..

[3] America S., Squadrone S., Brizio P., Stella C., Mantia M., Pederiva S., Brusa F., Mogliotti P., Garrone A., Abete M.C. (2020). Trace Elements and Rare Earth Elements in Honeys from the Balkans, Kazakhstan, Italy, South America, and Tanzania. Environ. Sci. Pollut. Res..

[4] Derewiaka D., Majewska E., Kuzak K., Szadkowska D. (2021). Comparison of Volatiles and Chemical Composition of Traditional and Non-Traditional Honey Available on the Polish Market. Appl. Sci..

[5] Isidorov V.A., Buczek K., Segiet A., Zambrowski G., Swiecicka I. (2018). Activity of Selected Plant Extracts against Honey Bee Pathogen *Paenibacillus larvae*. Apidologie.

[6] Isidorov V.A., Maslowiecka J., Pellizzer N., Miranda D., Bakier S. (2023). Chemical Composition of Volatile Components in the Honey of Some Species of Stingless Bees. Food Control.

[7] da Silva P.L., de Lima L.S., Caetano Í.K. (2017). Comparative Analysis of the Volatile Composition of Honeys from Brazilian Stingless Bees by Static Headspace GC—MS. Food Res. Int..

[8] Machado A.M., Miguel M.G., Vilas-Boas M., Figueiredo A.C. (2020). Honey Volatiles as a Fingerprint for Botanical Origin—A Review on Their Occurrence on Monofloral Honeys. Molecules.

[9] Patrignani M., Ciappini M.C., Tananaki C., Fagúndez G.A., Thrasyvoulou A., Lupano C.E. (2018). Correlations of Sensory Parameters with Physicochemical Characteristics of Argentinean Honeys by Multivariate Statistical Techniques. Int. J. Food Sci. Technol..

[10] Patrignani M., Fagúndez G.A., Tananaki C., Thrasyvoulou A., Lupano C.E. (2018). Volatile Compounds of Argentinean Honeys: Correlation with Floral and Geographical Origin. Food Chem..

[11] Tahir H.E., Xiaobo Z., Zhihua L., Yaodi Z. (2015). Comprehensive Evaluation of Antioxidant Properties and Volatile Compounds of Sudanese Honeys. J. Food Biochem..

[12] Tahir H.E., Xiaobo Z., Xiaowei H., Jiyong S., Mariod A.A. (2016). Discrimination of Honeys Using Colorimetric Sensor Arrays, Sensory Analysis and Gas Chromatography Techniques. Food Chem..

[13] Kružík V., Grégrová A., Rajchl A., Čížková H. (2017). Study on Honey Quality Evaluation and Detection of Adulteration by Analysis of Volatile Compounds. J. Apic. Sci..

[14] Taleuzzaman M., Alam M.J., Kala C., Rahat I. (2020). Honey of Authenticity: An Analytical Approach.

[15] Tanleque-Alberto F., Juan-Borrás M., Escriche I. (2019). Quality Parameters, Pollen and Volatile Profiles of Honey from North and Central Mozambique. Food Chem..

[16] Manyi-Loh C.E., Ndip R.N., Clarke A.M. (2011). Volatile Compounds in Honey: A Review on Their Involvement in Aroma, Botanical Origin Determination and Potential Biomedical Activities. Int. J. Mol. Sci..

[17] Manyi-Loh C.E., Clarke A.M., Ndip R.N. (2011). Identification of Volatile Compounds in Solvent Extracts of Honeys Produced in South Africa. Afr. J. Agric. Res..

[18] Moniruzzaman M., Rodríguez I., Ramil M., Cela R., Sulaiman S.A., Gan S.H. (2014). Assessment of Gas Chromatog-raphy Time-of-Flight Accurate Mass Spectrometry for Identification of Volatile and Semi-Volatile Compounds in Honey. Talanta.

[19] Tornuk F., Karaman S., Ozturk I., Toker O.S., Tastemur B., Sagdic O., Dogan M., Kayacier A. (2013). Quality Characterization of Artisanal and Retail Turkish Blossom Honeys: Determination of Physicochemical, Microbiological, Bioactive Properties and Aroma Profile. Ind. Crops Prod..

[20] Yamani H., Mantri N., Morrison P.D., Pang E. (2014). Analysis of the Volatile Organic Compounds from Leaves, Flower Spikes, and Nectar of Australian Grown Agastache Rugosa. BMC Complement. Altern. Med..

[21] Machado A.M., Miguel M.G., Vilas-boas M., Figueiredo A.C. (2021). Volatile Profile of Portuguese Monofloral Honeys: Significance in Botanical Origin Determination. Molecules.

[22] Devi A., Jangir J., Appaiah K.A. (2018). Chemical Characterization Complemented with Chemometrics for the Botanical Origin Identification of Unifloral and Multifloral Honeys from India. Food Res. Int..

[23] Missio P., Gauche C., Gonzaga L.V., Carolina A., Costa O. (2016). Honey: Chemical Composition, Stability and Authenticity. Food Chem..

[24] Costa A.C.V.D., Sousa J.M.B., da Silva M.A.A.P., Garruti D.D.S., Madruga M.S. (2018). Sensory and Volatile Profiles of Monofloral Honeys Produced by Native Stingless Bees of the Brazilian Semiarid Region. Food Res. Int..

[25] da Costa A.C.V., Sousa J.M.B., Bezerra T.K.A., da Silva F.L.H., Pastore G.M., da Silva M.A.A.P., Madruga M.S. (2018). Volatile Profile of Monofloral Honeys Produced in Brazilian Semiarid Region by Stingless Bees and Key Volatile Compounds. LWT.

[26] Feng S., Huang M., Crane J.H., Wang Y. (2018). Characterization of Key Aroma-Active Compounds in Lychee (*Litchi Chinensis* Sonn.). J. Food Drug Anal..

[27] Bertelsen A.S., Mielby L.A., Alexi N., Byrne D.V., Kidmose U. (2020). Sweetness Enhancement by Aromas: Measured by Descriptive Sensory Analysis and Relative to Reference Scaling. Chem. Senses.

[28] Đorđević S., Nedić N., Pavlović A., Milojković-Opsenica D., Tešić Ž., Gašić U. (2022). Honey with Added Value—Enriched with Rutin and Quercetin from Sophora Flower. J. Herb. Med..

[29] Grace E., Olarte Mantilla S.M., Sunarharum W.B., Ong C.M., Waanders J., D’Arcy B.R., Smyth H.E. (2020). Sensory Properties of Yellow Pea and Macadamia Honeys from Conventional and Flow Hive Extraction Methods. J. Sci. Food Agric..

[30] Mongi R.J., Ruhembe C.C. (2024). Sugar Profile and Sensory Properties of Honey from Different Geographical Zones and Botanical Origins in Tanzania. Heliyon.

[31] Praseptiangga D., Ferichani I.P., Mufida N. (2022). Development and Characterization of Bioactive Edible Films Based on Semi-Refined Kappa Carrageenan Incorporated with Honey and Kaempferia Galanga L. Essential Oil. Trends Sci..

[32] Romero C.A., Sosa N., Vallejos O.A., Navarro A.S., Yamul D.K., Baldi Coronel B.M. (2024). Physicochemical, Microbiological, and Sensory Properties of Stingless Bee Honey from Argentina. J. Apic. Res..

[33] Šedík P., Kňazovická V., Horská E., Kačániová M. (2018). Consumer Sensory Evaluation of Honey across Age Cohorts In Slovakia. Potravin. Slovak J. Food Sci..

[34] Šedík P., Horská E., Ivanišová E., Kačániová M., Krasnodȩbski A. (2019). Consumer Behaviour of Young Generation in Slovakia towards Cocoa-Enriched Honey. Potravin. Slovak J. Food Sci..

[35] Predanócyová K., Šedík P. (2024). Honey Market Challenges: Flavored Honey As Healthy Food Choice For Consumers. J. Microbiol. Biotechnol. Food Sci..

[36] Karabagias I.K., Louppis A.P., Karabournioti S., Kontakos S., Papastephanou C., Kontominas M.G. (2017). Characterization and Geographical Discrimination of Commercial *Citrus* Spp. Honeys Produced in Different Mediterranean Countries Based on Minerals, Volatile Compounds and Physicochemical Parameters, Using Chemometrics. Food Chem..

[37] Karabagias I.K., Dimitriou E., Halatsi E., Nikolaou C. (2017). Volatile Profile, Pigment Content, and in Vitro Radical Scavenging Activity of Flower, Thyme, and Fir Honeys Produced in Hellas. J. Food Chem. Nanotechnol..

[38] Louppis A.P., Karabagias I.K., Kontakos S., Kontominas M.G., Papastephanou C. (2017). Botanical Discrimination of Greek Unifloral Honeys Based on Mineral Content in Combination with Physicochemical Parameter Analysis, Using a Validated Chemometric Approach. Microchem. J..

[39] da Silva P.M., Gonzaga L.V., de Azevedo M.S., Biluca F.C., Schulz M., Costa A.C.O., Fett R. (2020). Stability of Volatile Compounds of Honey during Prolonged Storage. J. Food Sci. Technol..

[40] Zhu M., Sun J., Zhao H., Wu F., Xue X., Wu L., Cao W. (2022). Volatile Compounds of Five Types of Unifloral Honey in Northwest China: Correlation with Aroma and Floral Origin Based on HS-SPME/GC–MS Combined with Chemometrics. Food Chem..

[41] Li Q., Yang S., Zhang R., Liu S., Zhang C., Li Y., Li J. (2022). Characterization of Honey Peach (*Prunus persica* (L.) Batsch) Aroma Variation and Unraveling the Potential Aroma Metabolism Mechanism through Proteomics Analysis under Abiotic Stress. Food Chem..

[42] Li H., Lang Y., Liu Z., Song M., Jiang A., Li N., Chen L. (2024). Dynamic Variation in the Aroma Characteristics of Rhus Chinensis Honey at Different Stages after Capping. Food Chem..

[43] Leoni V., Giupponi L., Pavlovic R., Gianoncelli C., Cecati F., Ranzato E., Martinotti S., Pedrali D., Giorgi A., Panseri S. (2021). Multidisciplinary Analysis of Italian Alpine Wildflower Honey Reveals Criticalities, Diversity and Value. Sci. Rep..

[44] Jerković I., Obradović M., Kuś P.M., Šarolić M. (2013). Bioorganic Diversity of Rare *Coriandrum sativum* L. Honey: Unusual Chromatographic Profiles Containing Derivatives of Linalool/Oxygenated Methoxybenzene. Chem. Biodivers..

[45] Prđun S., Flanjak I., Svečnjak L., Primorac L., Lazarus M., Orct T., Bubalo D., Bilić Rajs B. (2022). Characterization of Rare Himalayan Balsam (Impatiens Glandulifera Royle) Honey from Croatia. Foods.

[46] Prđun S., Kremer D., Bubalo D., Svečnjak L. (2020). Physico-Chemical, Melissopalynological and Sensory Characteristics Osatsuma Mandarin Honey (*Citrus unshiu* Marc.)|Fizikalno-Kemijska, Melisopalinološka i Senzorska Svojstva Meda Od Unšijske Mandarine (*Citrus unshiu* Marc.). J. Cent. Eur. Agric..

[47] Arthur C.L., Pawliszyn J. (1990). Solid Phase Microextraction with Thermal Desorption Using Fused Silica Optical Fibers. Anal. Chem..

[48] Kamgang Nzekoue F., Angeloni S., Caprioli G., Cortese M., Maggi F., Marconi U.M.B., Perali A., Ricciutelli M., Sagratini G., Vittori S. (2020). Fiber-Sample Distance, An Important Parameter to Be Considered in Headspace Solid-Phase Microextraction Applications. Anal. Chem..

[49] Silva P., Freitas J., Nunes F.M., Câmara J.S. (2021). Effect of Processing and Storage on the Volatile Profile of Sugarcane Honey: A Four-Year Study. Food Chem..

[50] Durmaz N.E., Anli E., Güçer Y., Artik N. (2020). Bazı Türk Çiçek Ballarının Uçucu Bileşenlerinin Katı Faz Mikroekstraksiyonu Ve Gaz Kromatografisi-Kütle Spektrometresi İle Analizi. Eur. J. Sci. Technol..

[51] Kasiotis K.M., Baira E., Iosifidou S., Manea-Karga E., Tsipi D., Gounari S., Theologidis I., Barmpouni T., Danieli P.P., Lazzari F. (2023). Fingerprinting Chemical Markers in the Mediterranean Orange Blossom Honey: UHPLC-HRMS Metabolomics Study Integrating Melissopalynological Analysis, GC-MS and HPLC-PDA-ESI/MS. Molecules.

[52] Zhao J., Du X., Cheng N., Chen L., Xue X., Zhao J., Wu L., Cao W. (2016). Identification of Monofloral Honeys Using HPLC-ECD and Chemometrics. Food Chem..

[53] Wei Q., Sun J., Guo J., Li X., Zhang X., Xiao F. (2023). Authentication of Chaste Honey Adulterated with High Fructose Corn Syrup by HS-SPME-GC-MS Coupled with Chemometrics. LWT.

[54] Jerkovic I., Kranjac M., Suste M., Kus P.M., Svecnjak L. (2015). Rhamnus Frangula Honey: Screening of Volatile Organic Compounds and Their Composition After Short-Term Heating. Chem. Nat. Compd..

[55] Jerković I., Kranjac M., Marijanović Z., Zekić M., Radonić A., Tuberoso C.I.G. (2016). Screening of Satureja Subspicata Vis. Honey by HPLC-DAD, GC-FID/MS and UV/VIS: Prephenate Derivatives as Biomarkers. Molecules.

[56] Jerković I., Tuberoso C.I.G., Baranović G., Marijanović Z., Kranjac M., Svečnjak L., Kuś P.M. (2015). Characterization of Summer Savory (*Satureja hortensis* L.) Honey by Physico-Chemical Parameters and Chromatograph-ic/Spectroscopic Techniques (GC-FID/MS, HPLC-DAD, UV/VIS and FTIR-ATR). Croat. Chem. Acta.

[57] Srinivasan A., Maruthavanan T., Mayildurai R., Ramasubbu A. (2021). GC–MS Investigations of VOCs in South Indian Honey Samples as Environmental Biomarkers. Environ. Monit. Assess..

[58] Kuś P.M., Jerković I., Marijanović Z., Tuberoso C.I.G. (2017). Screening of Polish Fir Honeydew Honey Using GC/MS, HPLC-DAD, and Physical-Chemical Parameters: Benzene Derivatives and Terpenes as Chemical Markers. Chem. Biodivers..

[59] Türk G., Şen K. (2021). Changes of Various Quality Characteristics and Aroma Compounds of Astragalus Honey Obtained from Different Altitudes of Adana-Turkey. J. Food Process. Preserv..

[60] Kaziur-Cegla W., Jochmann M.A., Molt K., Bruchmann A., Schmidt T.C. (2022). In-Tube Dynamic Extraction for Analysis of Volatile Organic Compounds in Honey Samples. Food Chem. X.

[61] Vazquez L., Celeiro M., Sergazina M., Dagnac T., Llompart M. (2021). Optimization of a Miniaturized Solid-Phase Microextraction Method Followed by Gas Chromatography Mass Spectrometry for the Determination of Twenty Four Volatile and Semivolatile Compounds in Honey from Galicia (NW Spain) and Foreign Countries. Sustain. Chem. Pharm..

[62] Petretto G.L., Urgeghe P.P., Mascia I., Fadda C., Rourke J.P., Pintore G. (2017). Stir Bar Sorptive Extraction Coupled with GC/MS Applied to Honey: Optimization of Method and Comparative Study with Headspace Extraction Techniques. Eur. Food Res. Technol..

[63] Hamilton-Amachree A., Odokwo E.O. (2022). GC-MS Analysis of the Volatile Constituents and Antioxidant Activity of the Crude Honey Residue from Takum Local Government Area of Taraba State, Nigeria. Afr. Sci. J..

[64] Wang X., Yan S., Zhao W., Wu L., Tian W., Xue X. (2023). Comprehensive Study of Volatile Compounds of Rare Leucosceptrum Canum Smith Honey: Aroma Profiling and Characteristic Compound Screening via GC–MS and GC–MS/MS. Food Res. Int..

[65] Li H., Liu Z., Song M., Jiang A., Lang Y., Chen L. (2024). Aromatic Profiles and Enantiomeric Distributions of Volatile Compounds during the Ripening of Dendropanax Dentiger Honey. Food Res. Int..

[66] Li H., Liu Z., Shuai M., Song M., Qiao D., Peng W., Chen L. (2024). Characterization of Evodia Rutaecarpa (Juss) Benth Honey: Volatile Profile, Odor-Active Compounds and Odor Properties. J. Sci. Food Agric..

[67] Wang X., Rogers K.M., Li Y., Yang S., Chen L., Zhou J. (2019). Untargeted and Targeted Discrimination of Honey Collected by Apis Cerana and Apis Mellifera Based on Volatiles Using HS-GC-IMS and HS-SPME-GC−MS. J. Agric. Food Chem..

[68] Schanzmann H., Augustini A.L.R.M., Sanders D., Dahlheimer M., Wigger M., Zech P.M., Sielemann S. (2022). Differentiation of Monofloral Honey Using Volatile Organic Compounds by HS-GCxIMS. Molecules.

[69] Vyviurska O., Chlebo R., Pysarevska S., Špánik I. (2016). The Tracing of VOC Composition of Acacia Honey During Ripening Stages by Comprehensive Two-Dimensional Gas Chromatography. Chem. Biodivers..

[70] Kuan Wei S., Kamarulzaman Raja Ibrahim R., Nizam Lani M., Bahri Abd Razak S. Identification and Quantification of Volatile Compounds Released by Stingless Honey Using Long Optical Path Infrared Spectroscopy. Proceedings of the 4th International Science Postgraduate Conference, ISPC 2016.

[71] Nur Ain Mohammed Hassan K., Kamarulzaman Raja Ibrahim R., Hani Jameela Sapingi H., Afiq Daniel Azmi M., Zakaria Z., Ihsan N. (2020). Quality of Stingless Bee Honey Based on Volatile Organic Compounds and Gas Released. Proceedings of the Journal of Physics: Conference Series.

[72] Panseri S., Borgonovo F., Guarino M., Chiesa L., Piana M.L., Rizzi R., Mortarino M. (2023). Monitoring Volatile Organic Compounds and Aroma Profile of *Robinia pseudoacacia* L. Honey at Different Storage Temperatures during Shelf Life. Foods.

[73] Mohd Ali M., Hashim N., Abd Aziz S., Lasekan O.O. (2020). Principles and Recent Advances in Electronic Nose for Quality Inspection of Agricultural and Food Products. Trends Food Sci. Technol..

[74] Xiao Y., Huang Y., Chen Y., Zhu M., He C., Li Z., Wang Y., Liu Z. (2022). Characteristic Fingerprints and Change of Volatile Organic Compounds of Dark Teas during Solid-State Fermentation with Eurotium Cristatum by Using HS-GC-IMS, HS-SPME-GC-MS, E-Nose and Sensory Evaluation. LWT.

[75] Merkle S., Kleeberg K., Fritsche J. (2015). Recent Developments and Applications of Solid Phase Microextraction (SPME) in Food and Environmental Analysis—A Review. Chromatography.

[76] Oymen B., Aşır S., Türkmen D., Denizli A. (2022). Determination of Multi-Pesticide Residues in Honey with a Modified QuEChERS Procedure Followed by LC-MS/MS and GC-MS/MS. J. Apic. Res..

[77] Nolvachai Y., Amaral M.S.S., Marriott P.J. (2023). Foods and Contaminants Analysis Using Multidimensional Gas Chromatography: An Update of Recent Studies, Technology, and Applications. Anal. Chem..

[78] Shen X., Wang H., Yao L., Sun M., Yu C., Feng T., Kang W. (2025). Volatile Compounds Analysis and Sensory Profiling of Xinjiang Ili Different Floral Source Honey via GC-O-MS, GC-IMS and GC × GC-TOF MS. J. Food Sci..

[79] Gerhardt N., Birkenmeier M., Schwolow S., Rohn S., Weller P. (2018). Volatile-Compound Fingerprinting by Headspace-Gas-Chromatography Ion-Mobility Spectrometry (HS-GC-IMS) as a Benchtop Alternative to ^1^H NMR Profiling for Assessment of the Authenticity of Honey. Anal. Chem..

[80] Zhang L., Peng X., Wu X., Tie X., Ma J., Yi Y., Chen Y., Li K., Hou A., Yang J. (2025). Characterization and decoding of volatile organic compounds in Moso bamboo shoots (*Phyllostachys edulis* L.) at different growth stages: A combined analysis by HS-GC-IMS, HS-SPME-GC–MS, E-nose, and molecular docking. Food Res. Int..

[81] Feng D., Wang J., He Y., Ji X.J., Tang H., Dong Y.M., Yan W.J. (2021). HS-GC-IMS detection of volatile organic compounds in Acacia honey powders under vacuum belt drying at different temperatures. Food Sci. Nutr..

[82] Wei G., Dan M., Zhao G., Wang D. (2023). Recent advances in chromatography-mass spectrometry and electronic nose technology in food flavor analysis and detection. Food Chem..

[83] Acevedo F., Torres P., Oomah B.D., de Alencar S.M., Massarioli A.P., Martín-Venegas R., Albarral-Ávila V., Burgos-Díaz C., Ferrer R., Rubilar M. (2017). Volatile and Non-Volatile/Semi-Volatile Compounds and in Vitro Bioactive Properties of Chilean Ulmo (*Eucryphia cordifolia* Cav.) Honey. Food Res. Int..

[84] Xagoraris M., Skouria A., Revelou P.K., Alissandrakis E., Tarantilis P.A., Pappas C.S. (2021). Response Surface Methodology to Optimize the Isolation of Dominant Volatile Compounds from Monofloral Greek Thyme Honey Using Spme-Gc-Ms. Molecules.

[85] Karabagias I.K. (2022). Headspace Volatile Compounds Fluctuations in Honeydew Honey during Storage at In-House Conditions. Eur. Food Res. Technol..

[86] Pattamayutanon P., Angeli S., Thakeow P., Abraham J., Disayathanoowat T., Chantawannakul P. (2017). Volatile Organic Compounds of Thai Honeys Produced from Several Floral Sources by Different Honey Bee Species. PLoS ONE.

[87] Karlidag S., Keskin M., Bayram S., Mayda N., Ozkok A. (2021). Honey: Determination of Volatile Compounds, Antioxidant and Antibacterial Activities. Czech J. Food Sci..

[88] Tedesco R., Scalabrin E., Malagnini V., Strojnik L., Ogrinc N., Capodaglio G. (2022). Characterization of Botanical Origin of Italian Honey by Carbohydrate Composition and Volatile Organic Compounds (VOCs). Foods.

[89] Da Silva B.R., D’Apolito C., Botelho S.C.C., Cavalheiro L., De Andrade E.A., Wobeto C. (2021). Volatile Compounds in Off-Odor Honey. Cienc. Rural.

[90] Manickavasagam G., Saaid M., Shafie M.H., Lim V., Mohd Rodhi A. (2024). Chemometrics Exploration of Monosaccharides, Sugar Acids, Stable Carbon Isotopes, and Volatile Organic Compounds in Malaysian Stingless Bee Honey from Different Geographical Origins. J. Iran. Chem. Soc..

[91] Samborska K., Jedlińska A., Wiktor A., Derewiaka D., Wołosiak R., Matwijczuk A., Jamróz W., Skwarczyńska-Maj K., Kiełczewski D., Błażowski Ł. (2019). The Effect of Low-Temperature Spray Drying with Dehumidified Air on Phenolic Compounds, Antioxidant Activity, and Aroma Compounds of Rapeseed Honey Powders. Food Bioproc. Technol..

[92] Kolayli S., Kazaz G., Özkök A., Keskin M., Kara Y., Demir Kanbur E., Ertürk Ö. (2023). The Phenolic Composition, Aroma Compounds, Physicochemical and Antimicrobial Properties of *Nigella sativa* L. (Black Cumin) Honey. Eur. Food Res. Technol..

[93] Yan S., Liu Y., Zhao W., Zhao H., Xue X. (2023). Chemical Markers of a Rare Honey from the Traditional Spice Plant Amomum Tsao–Ko Crevost et Lemarié, via Integrated GC–MS and LC-MS Approaches. Food Res. Int..

[94] Robotti E., Campo F., Riviello M., Bobba M., Manfredi M., Mazzucco E., Gosetti F., Calabrese G., Sangiorgi E., Marengo E. (2017). Optimization of the Extraction of the Volatile Fraction from Honey Samples by SPME-GC-MS, Experimental Design, and Multivariate Target Functions. J. Chem..

[95] Svečnjak L., Jović O., Prđun S., Rogina J., Marijanović Z., Car J., Matošević M., Jerković I. (2019). Influence of Beeswax Adulteration with Paraffin on the Composition and Quality of Honey Determined by Physico-Chemical Analyses, 1 H NMR, FTIR-ATR and HS-SPME/GC–MS. Food Chem..

[96] Xagoraris M., Chrysoulaki F., Revelou P.K., Alissandrakis E., Tarantilis P.A., Pappas C.S. (2021). Unifloral Autumn Heather Honey from Indigenous Greek *Erica manipuliflora* Salisb.: Spme/Gc-Ms Characterization of the Volatile Fraction and Optimization of the Isolation Parameters. Foods.

[97] Özkök A., Sorkun K., Salih B. (2016). The Microscopic and GC-MS Analysis of Turkish Honeydew (Pine) Honey Türk Çam Ballarının Mikroskobik ve GC-MS Analizi. Hacet. J. Biol. Chem..

[98] Breschi C., Ieri F., Calamai L., Miele A., D’Agostino S., Melani F., Zanoni B., Mulinacci N., Cecchi L. (2024). HS-SPME-GC-MS Analysis of the Volatile Composition of Italian Honey for Its Characterization and Authentication Using the Genetic Algorithm. Separations.

[99] Damayanti N.W., Sahlan M., Lischer K., Hermansyah H., Kusumoputro B., Pratami D.K. (2019). Comparison of Original Honey (*Apis *sp. and *Tetragonula *sp.) and Fake Honey Compounds in Indonesia Using Gas Chromatography-Mass Spectrometry (GC-MS). AIP Conf. Proc..

[100] Karabagias I.K. (2022). HS-SPME/GC-MS Metabolomic Analysis for the Identification of Exogenous Volatile Metabolites of Monofloral Honey and Quality Control Suggestions. Eur. Food Res. Technol..

[101] Karabagias I.K., Badeka A., Kontominas M.G. (2020). A Decisive Strategy for Monofloral Honey Authentication Using Analysis of Volatile Compounds and Pattern Recognition Techniques. Microchem. J..

[102] Karabagias I.K., Karabagias V.K., Nayik G.A., Gatzias I., Badeka A.V. (2022). A Targeted Chemometric Evaluation of the Volatile Compounds of Quercus Ilex Honey in Relation to Its Provenance. LWT.

[103] Baroni M.V., Nores M.L., Díaz M.D.P., Chiabrando G.A., Fassano J.P., Costa C., Wunderlin D.A. (2006). Determination of Volatile Organic Compound Patterns Characteristic of Five Unifloral Honey by Solid-Phase Microextraction-Gas Chromatography-Mass Spectrometry Coupled to Chemometrics. J. Agric. Food Chem..

[104] Fallico B., Arena E., Zappala M. (2008). Degradation of 5-Hydroxymethylfurfural in Honey. J. Food Sci..

[105] Luca L., Pauliuc D., Oroian M. (2024). Honey Microbiota, Methods for Determining the Microbiological Composition and the Antimicrobial Effect of Honey—A Review. Food Chem. X.

[106] Khassenbekova Z., Gulayev A., Akhmetova A., Sergazy S., Baidildinova G., Kushugulova A., Kozhakhmetov S., Urasova M., Nurgozhin T. THE ANTIMICROBIAL ACTIVITY OF HONEY KAZAKHSTAN REGIONS. Proceedings of the Third International Scientific Conference.

[107] Wang Y., Gou X., Yue T., Ren R., Zhao H., He L., Liu C., Cao W. (2021). Evaluation of Physicochemical Properties of Qinling Apis Cerana Honey and the Antimicrobial Activity of the Extract against Salmonella Typhimurium LT2 in Vitro and in Vivo. Food Chem..

[108] Profile V., Determination N.V. (2019). Volatile Profile and Physico-Chemical Analysis of Acacia Honey for Geographical Origin And Nutritional Value Determination. Foods.

[109] Iwaniuk P., Kaczy P. (2021). Journal of Food Composition and Analysis Analysis of 22 Free Amino Acids in Honey from Eastern Europe and Central Asia Using LC-MS/MS Technique without Derivatization Step. J. Food Compos. Anal..

[110] Microextraction H.S., Mamedova M., Alimzhanova M.B. (2023). Determination of Biomarkers in Multifloral Honey by Vacuum-Assisted Determination of Biomarkers in Multifloral Honey by Vacuum Assisted Headspace Sol-id Phase Microextraction. Food Anal. Methods.

[111] Liang D., Wen H., Zhou Y., Wang T., Jia G., Cui Z., Li A. (2023). Simultaneous Qualitative and Quantitative Analyses of Volatile Components in Chinese Honey of Six Botanical Origins Using Headspace Solid-Phase Microextraction and Gas Chromatography–Mass Spectrometry. J. Sci. Food Agric..

[112] Wang X., Yang S., He J., Chen L., Zhang J., Jin Y., Zhou J., Zhang Y. (2019). A Green Triple-Locked Strategy Based on Volatile-Compound Imaging, Chemometrics, and Markers to Discriminate Winter Honey and Sapium Honey Using Headspace Gas Chromatography-Ion Mobility Spectrometry. Food Res. Int..

[113] Mekious S., Masseaux C., Loucif-Ayad W., Vercelli M. (2022). Pollen Quality and Sensory Attributes of Algerian Jujube (*Ziziphus lotus* (L.) Lam.) Honeys|Kakovost Peloda in Senzorične Lastnosti Medu Iz Alžirske Vrste Čičimaka (*Ziziphus lotus* (L.) Lam.). Acta Agric. Slov..

[114] Izabely Nunes Moreira F., de Medeiros L.L., de Carvalho L.M., Souza Olegario L., de Sousa Galvão M., da Franca S.A.M., Kênia Alencar Bezerra T., dos Santos Lima M., Suely Madruga M. (2023). Quality of Brazilian Stingless Bee Honeys: Cephalotrigona Capitata/Mombucão and Melipona Scutellaris Latrelle/Uruçu. Food Chem..

[115] Wang X., Lim L.T., Tan S., Fu Y. (2019). Investigation of the Factors That Affect the Volume and Stability of Espresso Crema. Food Res. Int..

[116] Kumar A., Gill J.P.S., Bedi J.S., Manav M., Ansari M.J., Walia G.S. (2018). Sensorial and Physicochemical Analysis of Indian Honeys for Assessment of Quality and Floral Origins. Food Res. Int..

[117] Alissandrakis E., Tarantilis P.A., Harizanis P.C., Polissiou M. (2007). Comparison of the Volatile Composition in Thyme Honeys from Several Origins in Greece. J. Agric. Food Chem..

[118] Ribeiro M.I., Fernandes A.J., Cabo P.S., Diniz F.J. (2019). Trends in Honey Purchase and Consumption in Trás-Os-Montes Region, Portugal. Econ. Reg..

[119] González M.M., De Lorenzo C., Pérez R.A. (2010). Development of a Structured Sensory Honey Analysis: Application to Artisanal Madrid Honeys. Food Sci. Technol. Int..

[120] He C., Liu Y., Liu H., Zheng X., Shen G., Feng J. (2020). Compositional Identification and Authentication of Chinese Honeys by 1H NMR Combined with Multivariate Analysis. Food Res. Int..

[121] Rahman M.M., Alam M.N., Fatima N., Shahjalal H.M., Gan S.H., Khalil M.I. (2017). Chemical Composition and Biological Properties of Aromatic Compounds in Honey: An Overview. J. Food Biochem..

[122] Iguchi H., Watanabe A. (2025). Honey Flavors Formed via Yeast Fermentation in Honey from Japanese Honeybees. Biosci. Biotechnol. Biochem..

[123] Yang H., Wu M., Shen X., Lai Y., Wang D., Ma C., Ye X., Sun C., Cao J., Sun C. (2025). A Comprehensive Review of VOCs as a Key Indicator in Food Authentication. eFood.

[124] Lapinskaitė V., Bartkutė P., Lukša-Žebelovič J., Strazdaitė-Žielienė Ž., Servienė E. (2025). Diversity and Functional Potential of Yeasts Inhabiting Honey Bee Drones. Microorganisms.

[125] Pokajewicz K., Lamaka D., Hudz N., Adamchuk L., Wieczorek P.P. (2024). Volatile Profile of Bee Bread. Sci. Rep..

[126] Zhang X.H., Gu H.W., Liu R.J., Qing X.D., Nie J.F. (2023). A Comprehensive Review of the Current Trends and Recent Advancements on the Authenticity of Honey. Food Chem. X.

[127] Pourmoradian A., Barzegar M., Gharaghani S., Sahari M.A. (2025). Honey Adulteration Detection Using the HS-SPME-IMS Technique Combined with Chemometric Analysis. Food Chem. X.

